# Characterization of an Experimental Two-Step Self-Etch Adhesive’s Bonding Performance and Resin-Dentin Interfacial Properties

**DOI:** 10.3390/polym13071009

**Published:** 2021-03-25

**Authors:** Abu Faem Mohammad Almas Chowdhury, Arefin Alam, Monica Yamauti, Pedro Álvarez Lloret, Pipop Saikaew, Ricardo Marins Carvalho, Hidehiko Sano

**Affiliations:** 1 Department of Restorative Dentistry, Graduate School of Dental Medicine, Hokkaido University, Kita 13, Nishi 7, Kita-ku, Sapporo 060-8586, Japan; arefin@den.hokudai.ac.jp (A.A.); myamauti@den.hokudai.ac.jp (M.Y.); sano@den.hokudai.ac.jp (H.S.); 2 Department of Conservative Dentistry and Endodontics, Sapporo Dental College and Hospital, Plot 24, Sector 8, Dhaka 1230, Bangladesh; 3 Department of Geology, Faculty of Geology, University of Oviedo, Jesús Arias de Velasco s/n, 33005 Oviedo, Spain; pedroalvarez@uniovi.es; 4 Department of Operative Dentistry and Endodontics, Faculty of Dentistry, Mahidol University, No. 6 Yothi Road, Ratchathewi District, Bangkok 10400, Thailand; pipop.sai@mahidol.ac.th; 5 Division of Biomaterials, Department of Oral Biological and Medical Sciences, Faculty of Dentistry, University of British Columbia, 2199 Wesbrook Mall, Vancouver, BC V6T 1Z3, Canada; rickmc@dentistry.ubc.ca

**Keywords:** dentin bonding, two-step self-etch adhesive, universal adhesive, microtensile bond strength, resin-dentin interface, scanning electron microscopy, ultra microhardness tester, elastic modulus

## Abstract

This study evaluated an experimental two-step self-etch adhesive (BZF-29, BZF) by comparing it with a reference two-step self-etch adhesive (Clearfil Megabond 2, MB) and a universal adhesive (G-Premio Bond, GP) for microtensile bond strength (μTBS) and resin-dentin interfacial characteristics. Twenty-four human third molars were used for the μTBS test. Bonded peripheral dentin slices were separated to observe the resin-dentin interface and measure the adhesive layer thickness with SEM. μTBS data of the central beams were obtained after 24 h and 6 months of water storage. Fracture modes were determined using a stereomicroscope and SEM. Nine additional third molars were used to determine the elastic modulus (E) employing an ultra microhardness tester. Water storage did not affect μTBS of the tested adhesives (*p* > 0.05). μTBS of BZF and MB were similar but significantly higher than GP (*p* < 0.05). BZF achieved the highest adhesive layer thickness, while GP the lowest. E of BZF and MB were comparable but significantly lower than GP (*p* < 0.05). Except for GP, the predominant fracture mode was nonadhesive. The superior bonding performance of BZF and MB could be attributed to their better mechanical property and increased adhesive thickness imparting better stress relief at the interface.

## 1. Introduction

Since their inception [1], one-step self-etching adhesives have been increasingly valued owing to their simplicity of application, reduced clinical application time, and ability to bond to various substrates. Incorporating the essential constituents with differing chemical properties in a single bottle, nevertheless, results in reduced shelf life, water sorption, phase separation, increased nano-leakage, and limited bond durability [2]. Consequently, the reported short-term [3,4] and long-term [5,6] laboratory and clinical performances [7,8] of two-step self-etch and three-step etch-and-rinse adhesives were found to be better than their one-step counterparts. The exception being the mildly acidic one-step self-etching adhesives, the annual failure rate of which is similar to that of two and three-step self-etch adhesives [9]. Based on the 2-hydroxyethylmethacrylate-free (HEMA) one-step self-tech adhesive technology developed by GC Corporation, a new two-step self-etch system has been proposed to improve adhesive-substrate interfaces’ longevity. Avoiding the use of HEMA would partially decrease the deleterious effect of materials hydrophilicity.

As a result of the constant and rapid development of adhesives, laboratory screening has become a crucial step to predict their clinical performance [10], constructed on the principle that the stronger the bond, the better it will withstand functional stress [11]. In this perspective, due to its excellent discriminative capability, standard operating procedure, profound use, and versatility, the microtensile bond strength (µTBS) test is considered the most suitable laboratory testing tool [12]. Moreover, the µTBS test has been recommended as the most stand-in in vitro assessment of composite resin restoration retention, particularly after subjecting the bonded specimens to a longevity test [13]. Besides the quantitative assessment of adhesion, valuation of the bonded interface’s morphologic characteristics achieved with electron microscopic observations adds additional qualitative insights to tooth-biomaterial interaction [14].

When applied to dentin, each adhesive creates a hybrid layer [15] that provides a stress-breaking effect when a load is applied [16]. A gradual transition of the structures’ mechanical properties across the resin-dentin interface influences the bond strength by relieving the stresses between the shrinking composite resin and the rigid dentin [17,18]. Therefore, an appreciation of the interfacial structures’ elastic modulus is crucial, commonly gained through the indentation method employing an ultra microhardness tester [17,18,19,20].

Analysis of the factors that simultaneously affect the bonding of adhesives to dentin is challenging. Different experimental approaches have been engaged to assess as many properties as possible. Still, most strategies fail to correlate the results as some of them are destructive, or the substrate originates from different teeth. This study used a same-tooth model to compare the 24 h and 6 months bond strength results and determine the adhesive-dentin interfacial characteristics aiming to exclude the confounding effects of teeth variability.

The purpose of this study was to evaluate the dentin bonding performance of an experimental two-step self-etch adhesive by comparing its µTBS to that of a reference two-step self-etch adhesive and a universal adhesive after 24 h and 6 months of water storage. In addition, the elastic modulus across the resin-dentin interfaces was evaluated using the indentation method, and micromorphology was characterized using scanning electron microscopy (SEM). The tested null hypotheses were (1) dentin bond strengths will not differ due to the adhesive, and (2) duration of water-storage, (3) elastic modulus of the structures across the resin-dentin interface, and (4) their micromorphological characteristics will not be different.

## 2. Materials and Methods

This investigation was conducted following the Declaration of Helsinki of 1975, revised in 2013. The Research Ethics Committee of Hokkaido University Graduate School of Dental Medicine approved the study (approval number 2018-7; approval date 1 February 2018). 

**Figure 1:**
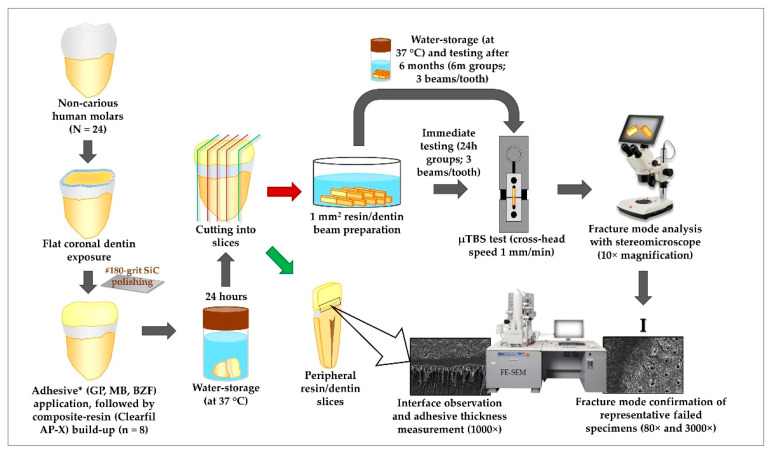
Schematic of specimen preparation and test set-ups for determining µTBS, fracture mode, and interface characterization. * GP, G-Premio Bond; MB, Clearfil Megabond 2; BZF, BZF-29.

All the human teeth used in this study were collected after the patients’ informed consent, stored in an aqueous solution of 0.5% Chloramine-T at 4 °C, and employed within six months of extraction. The whole study design is schematically presented in Figure 1.

### 2.1. Teeth Selection, Preparation, and Bonding Procedures

Twenty-four extracted sound human molars free of any signs of caries, cracks, or fractures were used for the µTBS test [21]. Flat, occlusal dentin surfaces were exposed using a gypsum model trimmer under water coolant and subsequently checked with a light-magnifier to confirm that no enamel remained on the surface. Exposed dentin surfaces were then prepared manually with 180-grit SiC paper (Sankyo-Rikagaku; Saitama, Japan) under running water for 60 s to produce clinically relevant smear layers [22]. The teeth were then randomly allocated to 3 groups (*n* = 8) for bonding with G-Premio Bond (GP; GC Corporation, Tokyo, Japan), Clearfil Megabond 2 (MB; Kuraray Noritake Dental Corporation, Nigata, Japan), and an experimental two-step self-etch adhesive, BZF-29 (BZF; GC Corporation, Tokyo, Japan). Each adhesive was applied according to the manufacturers’ instruction (Table 1) and light-cured at ≥1200 mW/cm^2^ (G-Light Prima-II plus, GC Corporation, Tokyo, Japan). After the application of adhesives, approximately 4 mm thick layers of composite resin (Clearfil AP-X, Kuraray Noritake Dental Inc., Niigata, Japan) were built up.

### 2.2. µTBS Test

After storage in distilled water at 37 °C for 24 h, each bonded tooth was sectioned into resin/dentin slices using a low-speed diamond saw (Isomet 1000, Buehler, Lake Bluff, IL, USA). Three central slices were selected and further cut into resin/dentin beams (cross-sectional area: 1 mm^2^) according to the non-trimming technique [21]. Bonded peripheral slices were separated to observe the resin/dentin interface with SEM. [25] Resin/dentin beams were then subjected to a microtensile bond strength (µTBS) test immediately (24 h) or after 6 months (6 m) of water storage. During the 6 months of storage time, the distilled water was changed weekly [26,27]. Twenty-four randomly selected beams originated from eight teeth were tested per group [21,23,28].

Each bonded beam was fixed to a Ciucchi’s jig with a cyanoacrylate adhesive (Model Repair II Blue, Dentsply-Sankin, Tokyo, Japan). They were then subjected to tensile force employing a 500-N load cell at a crosshead speed of 1 mm/min in a desktop testing apparatus (EZ-S, Shimadzu Co., Kyoto, Japan) until they were fractured. Each beam was tested within 5 min after removal from water storage to prevent sample drying [29]. The tensile load causing fracture of each beam was recorded and divided with the cross-sectional area to achieve the µTBS in megaPascals (MPa). The mean bond strength of three beams derived from each tooth represented the µTBS of that tooth, generating 8 values for each tested group.

### 2.3. Fracture Mode Analysis

After the μTBS test, the two ends of the fractured specimens were examined with 10× magnification using a stereomicroscope. Fracture modes at the dentin sides of the specimens were taken into consideration and classified into the following categories: adhesive failure, cohesive failure in dentin, cohesive failure in composite resin, and mixed failure [30]. In order to simplify the explanation of the results, the fractured beams showing cohesive and mixed failures were further combined into a nonadhesive failure category [31].

Selected representative fractured cases were further confirmed using a field emission scanning electron microscope (SEM; S-4800, Hitachi, Tokyo, Japan) at an accelerating voltage of 10 kV. The fractured specimens were carefully removed from the jig and mounted on an aluminum stub. They were coated with Pt-Pd using an ion sputtering device (E-1030, Hitachi, Tokyo, Japan) for 150 s and then observed with SEM. The fracture modes were determined with low magnification (80×). The specific features of fractured surfaces were further observed at higher magnification (3000×).

### 2.4. Interface Observation through SEM

Three bonded peripheral dentin slices (1 slice/adhesive) were employed for interface observation using SEM at an accelerating voltage of 10 kV. The specimens were prepared following a protocol described by Ting et al. [32]. The slices’ internal surfaces were sequentially polished with the waterproof SiC papers (600-, 800-, and 1000-grit) under running water and then with 6-, 3-, and 1-μm diamond pastes (DP-Paste; Struers, Denmark). After polishing, the specimens were cleaned with an ultrasonic device. They were then treated sequentially with 1 M hydrochloric acid for 30 s and 5% sodium hypochlorite solution for 5 min, followed by rinsing with water. The specimens were then room dried for 24 h. Finally, the samples were coated with Pt-Pd for 150 s and then observed with SEM at 1000× magnification. For the purpose of standardization, the adhesive layer thickness was measured at three spots of each bonded dentin slice: left lateral, central, and right lateral. The left and right lateral spots were determined at 500 μm mesially from each bonded slice’s left and right margins, respectively. At least 3 measurements were taken from each spot, and the mean of those values was considered the adhesive layer’s thickness for that slice. 

### 2.5. Specimen Preparation for Elastic Modulus Test

Nine additional bonded dentin slices (1 slice/tooth) were used for the elastic modulus (E) test employing indentation procedures. Nine additional molars were prepared and bonded with the tested adhesives (3 teeth/adhesive) in the same manner as mentioned above. After water storage (37 °C for 24 h), the bonded teeth were cut with a low-speed diamond saw (IsoMet 1000, Buehler) perpendicular to the bonded surface to obtain resin-dentin slices.

**Figure 2:**
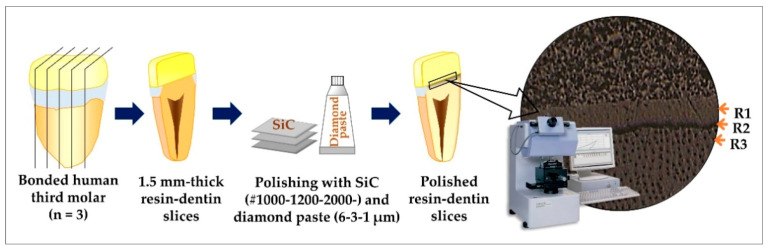
Schematic explaining the specimen preparation methodology and test set-up for determining elastic modulus. The resin-dentin interface was microscopically divided into three target regions—the adhesive layer (**R1**), adhesive-dentin interface (**R2**), and sound dentin (**R3**).

As shown in Figure 2, one central slice from each tooth was selected, prepared, and tested for E following a protocol described by Chowdhury et al. [33]. Each bonded dentin slice was sequentially finished with 1000-, 1200-, and 2000-grit waterproof SiC paper under running water and polished with 6-, 3-, and 1-µm diamond pastes for 1 min each. The specimens were cleaned with an ultrasonic unit with distilled water for 3 min after each finishing and polishing step.

### 2.6. Indentation Tests for Elastic Modulus (E)

E across the adhesive-dentin interface was measured with a dynamic ultra-micro-hardness tester (DUH-211, Shimadzu; Figure 2). The device contained a triangular pyramidal diamond indenter with a tip angle of 115° and a radius of 0.1 μm. All the specimens were tested at ambient temperatures (22 °C–24 °C) with a maximum humidity of 30%. Three regions—the adhesive layer, adhesive-dentin interface, and sound dentin—were targeted. All indentations were performed at a constant speed of 0.2926 mN/s and held for 10 s at peak load [33]. The maximum loads employed were 5.05 mN. E values were obtained from the default software of the testing device. At least a 10 μm distance between adjacent indentations was maintained to avoid the influence of the residual stress from adjacent indentations. Poisson’s ratio was 0.30.

### 2.7. Statistical Analysis

The normality and homogeneity of all data were checked using the Shapiro‒Wilk test and Levene’s test. Based on those test results, two-way ANOVA followed by Tukey’s test, Welch ANOVA followed by the Games-Howell test, and Kruskal‒Wallis followed by the Dunn‒Bonferroni test was employed to analyze the µTBS, adhesive thickness, and E data, respectively (*α* = 0.05). All statistical analyses were done using SPSS 25.0 for Windows (SPSS, Chicago, IL, USA).

## 3. Results

### 3.1. µTBS Test

No pretest bond failure was encountered in this study. The µTBS test results are summarized in Table 2. Two-way ANOVA revealed significant effects of adhesives (*F* = 46.952, *p* < 0.001) on the µTBS, but the effects of water storage duration was not significant (*F* = 2.807, *p* = 0.101). In addition, the two-way interaction between these variables was not statistically significant (*F* = 0.373, *p* = 0.691).

### 3.2. Fracture Modes

The percentage of the fracture modes observed in this study is shown in Table 2. For BZF and MB, the predominant failure mode in 24 h groups was nonadhesive (79%). The percentage of adhesive failures increased in 6 m groups, showing adhesive failure in almost half of the beams (46%). For GP, the failure mode was only adhesive (100%) in 24 h. However, in the 6 m group, 37% of the beams showed nonadhesive failure. No cohesive failure in composite resin was observed in this study.

**Figure 3:**
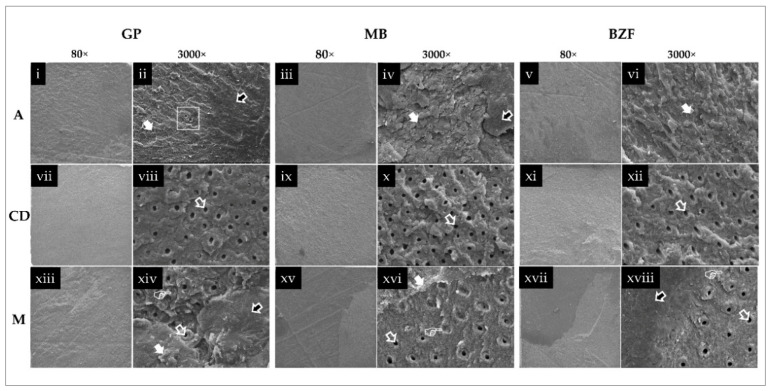
Representative SEM images of the adhesives’ (GP—G-Premio Bond; MB—Clearfil Megabond 2; BZF—BZF 29) fracture modes. The images under 3000× columns demonstrate the magnified specific features of the preceding 80× images. Row A is adhesive failure, row CD is cohesive failure in dentin, and row M is the mixed type of failure involving adhesive and dentin. The white arrows indicate a cohesive failure in the adhesive (**ii**,**iv**,**vi**); the white rectangle includes bubbles (**ii**); white-bordered black arrows show darker adhesive areas indicating failure at the composite resin-adhesive interface (**ii**,**iv**,**xiv**,**xviii**); transparent white arrows indicate the dentinal tubules’ openings (**viii**,**x**,**xii**,**xiv**,**xvi**,**xviii**). The mixed failure patterns (**xiv**,**xvi**,**xviii**) also show complete or partially occluded dentinal tubules (white hands).

Representative SEM images of the fracture modes of selected failed specimens are shown in Figure 3.

When representative fractured specimens were examined through SEM at higher magnifications (3000×), bubbles could be seen in the adhesive failure category of GP (Figure 3ii). Similar specimens of MB (Figure 3iv) and BZF (Figure 3vi) were devoid of such features.

### 3.3. Interface Observation

**Figure 4:**
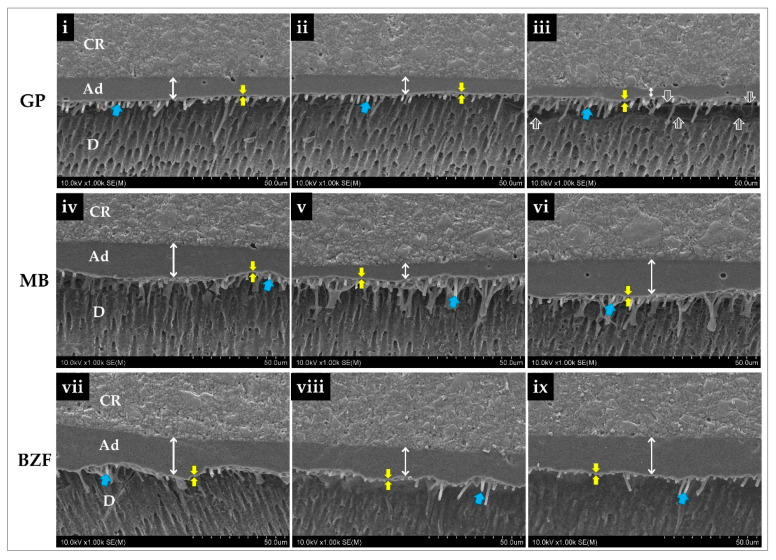
Representative SEM images of interfacial structures (1000×) of the tested adhesives—GP (G-Premio Bond), MB (Clearfil Megabond 2), and BZF (BZF-29). Double-ended white arrows represent the extension of the adhesive layers measured at left lateral (**i**,**iv**,**vii**), central (**ii**,**v**,**viii**), and right lateral (**iii**,**vi**,**ix**) spots of each bonded dentin slice. The electron-lucent area between the twin yellow arrows demarcates the hybrid layer. Blue arrows indicate resin tags. The striped arrows show gap formation at the resin-dentin interface (**iii**). CR—composite resin; Ad—adhesive layer; D—dentin.

Representative SEM images of each resin-dentin interface captured at the left lateral, central, and right lateral spots of each bonded dentin slice are demonstrated in Figure 4.

Hybrid layers could be seen clearly in all the groups. The interfaces of MB showed more extended resin tags compared to those of GP and BZF. The number of resin tags was most in MB. In general, BZF showed more uniform adhesive layer thickness than the rest. Interfacial gaps were observed between adhesive and dentin in some parts of GP. The mean thicknesses of the adhesive layers of GP, MB, and BZF were 8.8 ± 2.6 µm, 13.5 ± 4.6 µm, and 18.4 ± 2.5 µm, respectively (Table 2). A Welch ANOVA demonstrated a significant difference among the adhesive layers’ thickness values (*F* = 42.134, *p* < 0.001). Multiple comparisons with a Games‒Howell test revealed that the adhesive layer thicknesses of MB and BZF were significantly higher than that of GP. At the same time, BZF was significantly thicker than MB (*p* < 0.05).

### 3.4. Elastic Modulus (E)

The means and standard deviations of E across the adhesive-dentin interface of the tested groups are shown in Table 3.

Kruskal‒Wallis tests revealed very strong evidence of differences (*p* < 0.001) between the mean ranks of at least one pair of tested groups within the adhesive layer and adhesive-dentin interface. Within the adhesive layer, Dunn’s pairwise test demonstrated that the mean elastic modulus of GP (7192.6 ± 133.8 MPa) was significantly higher (*p* < 0.05, adjusted using Bonferroni correction) than MB (5730.9 ± 186.1 MPa) and BZF (5852.7 ± 97.1 MPa). There was no evidence of a difference between MB and BZF (*p* > 0.05).

**Figure 5:**
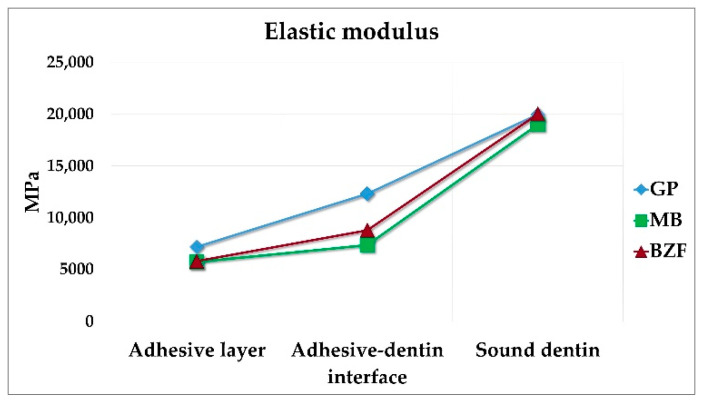
The graph shows a gradually increasing elastic modulus (E) across the adhesive-dentin interface. G-Premio Bond (GP), Clearfil Megabond 2 (MB), and BZF-29 (BZF).

Similarly, within the adhesive-dentin interface, Dunn’s pairwise test demonstrated that the mean elastic modulus of the GP group (12,314.4 ± 975.2 MPa) was significantly higher (*p* < 0.05, adjusted using Bonferroni correction) than MB (7372.0 ± 169.7 MPa) and BZF groups (8798.8 ± 1090.4 MPa). There was no evidence of a difference between MB and BZF groups (*p* > 0.05). The adhesive brand did not influence the E values of sound dentin. A gradual increase of E values was apparent in all the groups, starting with the lowest values in the softer adhesive layer and rising through the harder adhesive-dentin interface to end with the highest values in relatively stiff dentin (Figure 5).

## 4. Discussion

Although most self-etch adhesive systems contain the same components, they can differ profoundly regarding these components’ proportional amount [34,35]. Consequently, specific variations related to the adhesive composition might be considered to justify their bonding effectiveness. Previous in vitro studies have reported that two-step self-etch adhesives show higher bond strengths to tooth tissues than one-step self-etch adhesives [36,37,38,39,40]. Two-step self-etch adhesives, which involve applying an additional layer of solvent-free hydrophobic resin, create stronger adhesive layers than one-step self-etch adhesives, which contain hydrophilic monomers, water, and volatile solvents [41]. Similarly, in the current investigation, our results showed that the type of tested adhesives (one-step and two-step) exerted significant effects on their microtensile bond strength (*p* < 0.001).

The one-step universal adhesive G-Premio Bond (GP) would contain more amount of water for acidic functional monomers’ dissociation to be effective in the self-etch approach than its two-step counterparts (MB and BZF) [42]. Moreover, it needs to be sufficiently hydrophilic to properly bond with “wet” dentin, yet at the same time, become as hydrophobic as possible once polymerized to prevent water sorption and hydrolysis over time. However, GP contains highly volatile acetone. Within GP’s shorter application time (10 s), acetone evaporates quickly, leaving water behind. Too much remaining water can contribute to incomplete polymerization and HEMA’s absence to phase separation, culminating in a weak interface and premature bond failure [23,43,44].

Clearfil Megabond 2 (Japanese version of Clearfil SE 2) is the improved successor of Clearfil SE Bond—the gold standard two-step self-etch adhesive [9]. Previous studies have also reported its high and stable bonding performance with different dentin substrates [22,24,33]. In the current investigation, besides due to its additional hydrophobic resin layer, MB’s superior bonding performance than GP (*p* < 0.05) could also be attributed to its new photoinitiator, which improves its degree of conversion, leading to enhanced mechanical properties, lower water sorption levels, and higher bond strengths [24].

The bonding performance of the experimental two-step self-etch adhesive BZF-29 (BZF) was comparable to MB (*p* > 0.05) but significantly higher than GP (*p* < 0.05; Table 2). The interfaces’ representative SEM images revealed that contrary to MB and BZF, GP showed interfacial gaps, indicating a weaker resin-dentin interface (Figure 4iii).

Because of the ability to produce densely cross-linked polymers, bisphenol A-glycidyl methacrylate (Bis-GMA), urethane dimethacrylate (UDMA), and triethylene glycol dimethacrylate (TEGDMA) are the three most frequently used hydrophobic dimethacrylates in dental adhesives, which directly provide mechanical strength to a cured resin [45]. Despite being hydrophobic, due to the polar-ether linkage and the hydroxyl groups, water sorption of these three dimethacrylate-containing adhesives is inevitable, where UDMA shows the least and TEGDMA shows the highest water sorption [46]. This could prove advantageous if TEGDMA were added to primer, where hydrophilicity is essential for resin infiltration, and UDMA to the bonding resin, preventing hydrolytic degradation. Bis-GMA is commonly used in dental adhesives and composite resins because of its higher molecular weight, lower polymerization shrinkage, and fast hardening ability [46]. However, such properties of Bis-GMA also lead to excessive viscosity, rigidity, and reduced conversion rate [47]. Therefore, UDMA and/or TEGDMA are incorporated as the ‘diluents’, which can reduce the viscosity and rigidity [45,46]. In addition, UDMA’s comparable molecular weight to Bis-GMA allows it to be used alone or in combination with TEGDMA in some adhesives [48].

GC Corporation recently marketed a new adhesive named ‘G2-BOND Universal’. Unlike their already existing popular universal adhesive G-Premio Bond (GP), G2-BOND Universal adhesive is a two-bottle system but has a HEMA-free composition similar to GP [49]. G2-BOND Universal has also been claimed to provide a more hydrophobic bond layer thanks to its two-bottle strategy and UDMA in the bonding resin [49,50]. According to the brochure, G2-BOND Universal can produce an optimally thick adhesive layer, imparting a better shock-absorbing effect against shrinkage stress [51].

It is probably reasonable to assume that BZF might have employed those dimethacrylates or their combinations. Our results showed that BZF achieved a significantly thicker adhesive layer than GP and MB (*p* < 0.05; Figure 4), which in turn improves fracture toughness, preventing cohesive failure in the adhesive layer. The presence of interfacial gaps in GP having a significantly thinner adhesive layer than MB and BZF supports this speculation. Previous studies also reported superior bond strengths with thicker adhesive layers, and the reason given was better stress distribution due to increased elasticity of the adhesive layer [52,53]. Interestingly, SEM images revealed that the adhesive layer thickness of BZF could get as high as 38 µm (approx.), noticeably thicker than MB and GP, which could be approximately 22 µm and 14 µm, respectively (Supplementary Figure S1). The results of statistical comparison drawn between the adhesive layers followed the same trend (Table 2). In this context, it is worth mentioning that the standardized thickness measurement method employed in this investigation targeted the same spots for all adhesives, not necessarily including the thickest spots.

Van Meerbeek et al. reported a significantly lower elastic modulus of the adhesive-dentin interface than that of unaltered dentin and a gradual increase, starting from a softer adhesive layer, followed by a continued increase at the relatively harder adhesive-dentin interface, ultimately reaching the highest values in the stiffer dentin [17]. The gradual transition of the mechanical properties across the resin-dentin area contributes to bond strength by relieving the stresses between the shrinking composite resin and the rigid dentin. The E results and the gradient of values across the resin-dentin area demonstrated in this study concur with this report (Table 3 and Figure 5) and further substantiate the bond strength results. According to our findings, the E of the adhesive layer and adhesive-dentin interface of MB and BZF was similar (*p* > 0.05) but significantly lower than those of GP (*p* < 0.05), indicating an inverse relationship with their bond strength. Freitas et al. reported similar observations with one-step and two-step self-etch adhesives and concluded that lower E yielded higher bond strength, imparting adequate resistance of the adhesive to elastic deformation under stress [54].

In the current study, water storage duration (24 h and 6 months) did not significantly affect the adhesives’ dentin bond strength (*p* = 0.101; Table 2). Previous studies also reported stable bond strength values after 6 months of water storage [54,55,56]. The high permeability of hybridized dentin formed by simplified adhesives [57,58,59,60,61,62], the separation of phases between hydrophilic and hydrophobic monomers [63], and the high quantities of solvents [44,64,65] are factors that decrease the durability of adhesive restorations. It would probably be reasonable to assume that the extent of water diffusion in the present study was probably not enough to degrade the tested adhesive-dentin interfaces in 6 months. More extended storage periods should be evaluated to confirm this speculation.

The 24 h and 6 months µTBS results of the adhesives tested in this study were further supplemented by their failure patterns (Table 2). In our previous investigation [22,33], we demonstrated that the improved interfacial mechanical property of the two-step self-etch adhesive MB resulted in significantly higher bond strengths and increased nonadhesive failure percentages compared to the one-step self-etch adhesive GP. The failure patterns of the present investigation showed similar results. Both two-step adhesives MB and BZF showed a predominance of nonadhesive failure (≥54%) in all storage durations, implying a stronger and more stable adhesive layer, leading to higher bond strengths. GP, having the weakest bond among the three, showed predominantly adhesive failures (≥63%).

According to the results of the present investigation, adhesive type influenced the bond strength. However, the effect of water storage duration on bond strength was not significant. Moreover, the adhesives’ elastic modulus and interfacial micromorphology were found to be material dependent, exerting a substantial influence on the adhesive performance. Therefore, except for the second, all null hypotheses had to be rejected. In addition to the dimethacrylates, the degree of filler loading could also modify the adhesives’ rheological properties [66]. Transmission electron microscopic assay and energy dispersive X-ray spectroscopy of the adhesive-dentin interface may add valuable insights in this aspect. Future studies should be aimed at addressing these issues.

## 5. Conclusions

The results of this study revealed that the performance of two-step self-etch adhesives BZF-29 and Clearfil Megabond 2 was comparable but superior to the tested one-step universal adhesive G-Premio Bond in terms of bond strength, interfacial mechanical property, and morphological characteristics. The thicker dimension of the adhesive layer of BZF-29 might have contributed to its high bond strength. The 6 month water storage time did not alter the tested adhesives’ bond strength.

## Figures and Tables

**Table 1:** Adhesive systems (Lot number), composition, and application procedures.

Adhesives (Lot No.)	Composition	Application Procedures as per Manufacturers’ Instructions
^€^ G-Premio Bond (1807031)	10-MDP, 4-META, 10-MDTP, methacrylate acid ester, distilled water, acetone, photoinitiators, fine powdered silica	1. Apply using a micro brush. 2. Leave undisturbed for 10 s. 3. Dry thoroughly with air under maximum air pressure. 4. Light cure for 10 s.
^đ^ Clearfil Megabond 2 (000095)	**Primer**: 10-MDP, HEMA, hydrophilic aliphatic dimethacrylate, dl-CQ, water **Bond**: 10-MDP, Bis-GMA, HEMA, dl-CQ, hydrophobic aliphatic dimethacrylate, initiators, accelerators, silanated colloidal silica	1. Apply the primer and leave for 20 s. 2. Gentle air-blowing for >5 s. 3. Apply the bond. 4. Gentle air-blowing to make the film uniform. 5. Light-cure for 10 s.
^æ^ BZF-29 (1907201G-primer) (1907172-bond)	**Primer**: 4-META, MDP, dimethacrylate, photoinitiator, water, acetone, silica, MDTP **Bond**: Dimethacrylate, photoinitiator, silica	1. Apply the primer and leave for 10 s. 2. Dry with moderate air-blow for 5 s. 3. Apply the bond. 4. Gentle air-blowing to make the film uniform. 5. Light-cure for 5 s.

^€^ Composition as reported by Saikaew et al. [23]; ^đ^ Composition as reported by Sato et al. [24]; ^æ^ Information as provided by the manufacturer; 10-MDP, 10-methacryloyloxydecyl dihydrogen phosphate; 4-META, 4-methacryloxyethyl trimellitic anhydride; 10-MDTP, 10-methacryloxydecyl dihydrogen thiophosphate; HEMA, 2-hydroxyethylmethacrylate; CQ, camphorquinone; Bis-GMA, bisphenol-A-diglycidyl methacrylate.

**Table 2:** The mean values ± standard deviations (SD) of adhesive layer thickness (in µm) and µTBS (in MPa; *n* = 8), and the percentage of fracture modes (A/CD/CC/M) *.

Adhesives	Adhesive Layer Thickness Mean ± SD	24 Hours (24 h)		6 Months (6 m)	
		µTBS ± SD	A/CD/CC/M	µTBS ± SD	A/CD/CC/M
G-Premio Bond (GP)	8.8 ± 2.6 A	39.0 ± 6.0 A	100/0/0/0	37.6 ± 5.0 A	63/29/0/8
Clearfil Megabond 2 (MB)	13.5 ± 4.6 B	55.6 ± 4.6 B	21/75/0/4	51.2 ± 3.9 B	46/50/0/4
BZF-29 (BZF)	18.4 ± 2.5 C	55.3 ± 5.7 B	21/29/0/50	53.5 ± 6.2 B	46/46/0/8

Values with different uppercase letters indicate statistically significant differences between tested groups. Tukey’s HSD test made multiple comparisons for the µTBS and Games‒Howell test for adhesive layer thickness (*p* < 0.05). * A, adhesive failure; CD, cohesive failure in dentin; CC, cohesive failure in composite resin; M, mixed failure. CD/CC/M, together, constituted the nonadhesive failure category [31].

**Table 3:** Mean elastic modulus ± standard deviation measured in MPa for different locations (*n* = 9 indentations/location/group).

Adhesives	Adhesive Layer	Adhesive-Dentin Interface	Sound Dentin *
G-Premio Bond	7192.6 ± 133.8 B	12,314.4 ± 975.2 B	19,975.6 ± 900.4
Clearfil Megabond 2	5730.9 ± 186.1 A	7372.0 ± 169.7 A	19,007.8 ± 922.8
BZF-29	5852.7 ± 97.1 A	8798.8 ± 1090.4 A	20,016.7 ± 1524.9

Values with different uppercase letters indicate statistically significant differences between groups within each location (Dunn‒Bonferroni test, *p* < 0.05). * No significant difference was observed between the groups.

## Data Availability

The data presented in this study are available on request from the corresponding author.

## Supplementary Materials

The supplementary figure is available online at https://www.mdpi.com/2073-4360/13/7/1009/s1.
