# Supplementary material for: Characterization of an Experimental Two-Step Self-Etch Adhesive’s Bonding Performance and Resin-Dentin Interfacial Properties
Source: Polymers (Basel). 2021 Mar 25;13(7):1009. doi: 10.3390/polym13071009 (PMC8036260; doi:10.3390/polym13071009)

Supplementary material:

Figure S1. Representative SEM images (1000×) of the resin-dentin interfaces showed areas where the adhesive thicknesses were highest. In the one-step universal adhesive G-Premio Bond, the maximum adhesive layer thickness observed was approximately 14 µm. The maximum thicknesses of the two-step self-etch adhesives, Clearfil Megabond 2 and BZF-29, reached around 22 µm and 38 µm, respectively. The double-ended white arrows indicate the extensions of the adhesive layers (in µm). CR, Composite resin; Ad, Adhesive; D, Dentin.

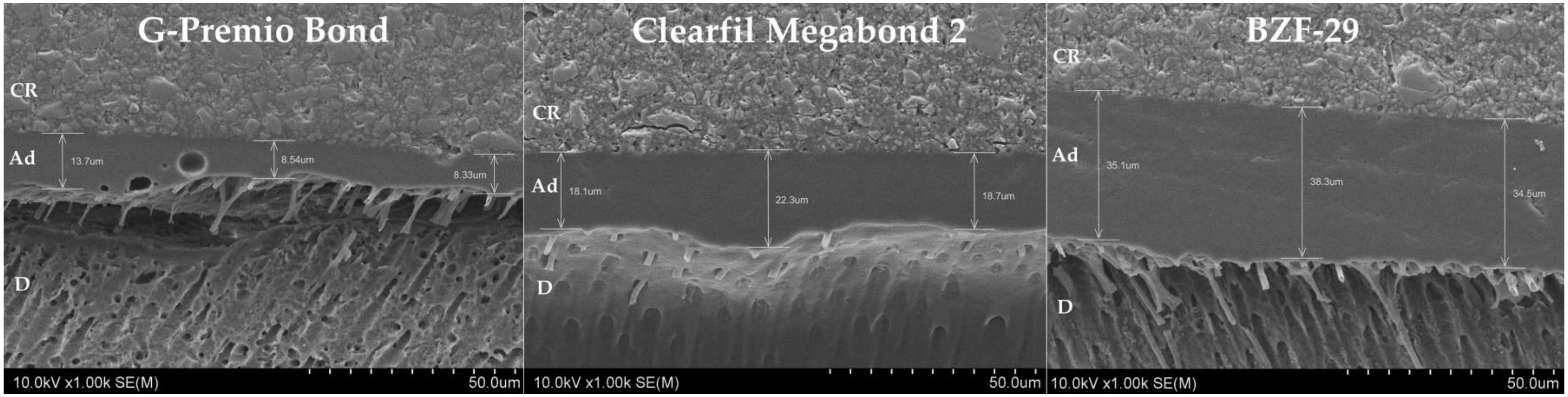

Supplement: Supplementary file 1 [file polymers-13-01009-s001.pdf]
